# Surgical Treatment of Radiation-Induced Brachial Plexus Neuropathy in Breast Cancer Patients after Adjuvant Radiotherapy: A Systematic Review

**DOI:** 10.1007/s43465-025-01540-0

**Published:** 2025-09-08

**Authors:** Carin Carroll, Anshumi Desai, Shelby Burks, Nathan Carberry, Kyle Xu, Kashyap Komarraju Tadisina

**Affiliations:** 1 Department of Obstetrics and Gynecology, University of Chicago, Chicago, IL USA; 2 Division of Plastic and Reconstructive Surgery, DeWitt Daughtry Family Department of Surgery, University of Miami Miller School of Medicine, Miami, FL USA; 3 Department of Neurological Surgery, University of Miami Miller School of Medicine, Miami, FL USA; 4 Department of Neurology, University of Miami Miller School of Medicine, Miami, FL USA

**Keywords:** Brachial neuritis, Radiation plexopathy, Breast cancer, Radiation

## Abstract

**Purpose:**

Radiotherapy is a frequently employed adjuvant treatment modality in breast cancer patients that carries debilitating side effects including radiation-induced brachial plexus neuritis (RIBPN). RIBPN is a neurological impairment that occurs following radiation exposure and causes pain, paresthesia, and weakness. The goal of this study is to review literature on surgical treatments for RIBPN and explore areas for further investigation.

**Methods:**

A comprehensive search of PubMed, SCOPUS and Embase databases was conducted using search terms related to RIBPN. The authors reviewed the titles, abstracts, and full texts to identify those that discussed surgical management of RIBPN in breast cancer patients. Out of 306 studies, 9 articles met inclusion criteria and were reviewed (Fig. 1).

**Findings:**

41 patients were treated surgically for RIBPN, with six distinct surgical techniques employed. External neurolysis was the most common treatment, benefiting 81.8% of patients with improvements in pain, although some patients experienced worsening motor strength. Techniques like segmental nerve resection with autografting and cervical thoracic laminectomy significantly enhanced pain and motor function. Other techniques including neurolysis with nerve transfer and gracilis-free muscle transfer provided varying degrees of recovery and pain relief.

**Conclusion:**

Our review highlights that while neurolysis, whether alone or combined with nerve transfer, free muscle transfer, or omentoplasty, and other methods including nerve grafting or dorsal root lesions offer surgical treatment options for RIBPN, these modalities are not well studied. Further investigation into the efficacy of these options and alternative surgical treatments may be warranted to improve outcomes among breast cancer patients.

**Supplementary Information:**

The online version contains supplementary material available at 10.1007/s43465-025-01540-0.

## Introduction

Radiation-induced brachial plexus neuritis (RIBPN) was first described in 1966 by Stoll and Andrews in their study on neurologic symptoms in patients who underwent radiation post-breast cancer surgery [1, 2]. RIBPN manifests as a neurological impairment, either transient or permanent, related to radiation affecting the brachial plexus. This condition is primarily a severe side effect of radiation to the breast, chest wall, and lymph nodes [3, 4]. Damage typically occurs from nodal radiation to the brachial plexus, leading to a pathological process of neural tissue fibrosis, resulting in direct and indirect nerve injury [3, 5, 6].

Symptoms of RIBPN, appearing between six months and several years post-radiation, include neuropathic pain, paresthesia, dysesthesia, numbness, lymphedema, and motor weakness [3]. Severe cases may progress to partial or complete limb paralysis [5]. It is currently estimated that between 1 and 9% of patients who have undergone radiation therapy to the supraclavicular and axillary lymph node regions suffer from RIBPN. Recent findings by Warade et al. indicate a 1.2% prevalence in radiated patients, with increasing prevalence as more breast cancer patients receive radiation [3, 7, 8].

**Fig. 1:**
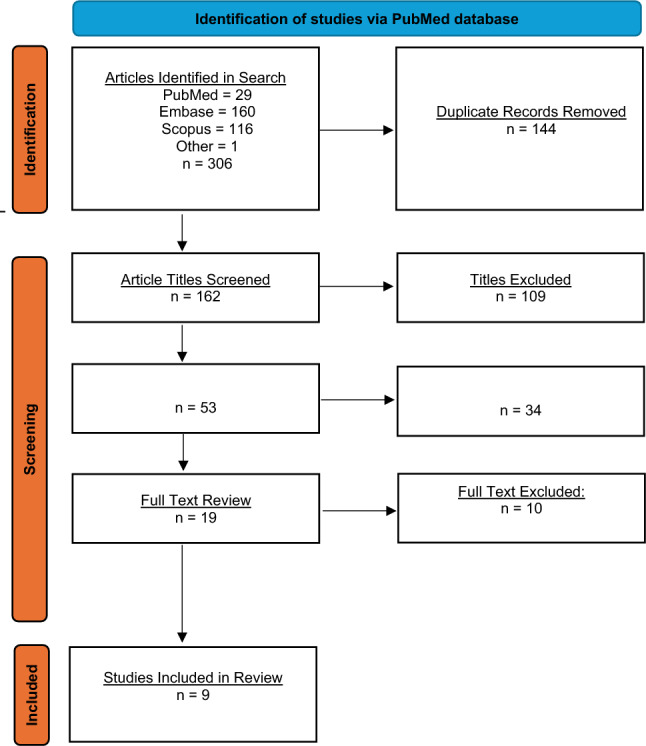
PRISMA flow chart

Treatment for mild brachial plexus injuries (grade 1 or 2) generally involves non-opioid painkillers, tricyclic antidepressants, benzodiazepines, anti-epileptics, and physical or occupational therapies [9, 10]. For severe injuries with substantial nerve damage, medical treatment is often insufficient, necessitating surgical intervention [11]. Current surgical treatment options for these patients are limited and include neurolysis, segmental excision and reconstruction, debridement and flap coverage, or a combination of the above [10, 11]. There remains a paucity of evidence and long-term data to effectively guide surgical interventions [12]. The authors seek to review existing literature regarding surgical treatment of RIBPN in breast cancer patients to help guide the management of these patients(see Fig. 1).

## Methods

A comprehensive search of PubMed, SCOPUS and Embase databases was conducted in January 2024 for terms related to RIBPN. The following search terms were used: “radiation neuritis breast” OR “radiation-induced brachial plexopathy” OR “surgical treatment of radiation neuritis breast” OR “surgical treatment of radiation-induced brachial plexopathy.” These terms were combined using the Boolean operator OR to maximize retrieval of relevant literature related to both the condition and its surgical management. The search was restricted to studies published between 1/2005 and 4/2023 and thus articles included in the review represent primary data on surgical treatments of RIBPN from 2005 to 2023 (Fig. 1 and Supplemental Fig. 1).

To systematically assess the included studies, two independent reviewers (CCC and AD) evaluated each article for eligibility based on predefined inclusion and exclusion criteria, with disagreements resolved by discussion or consensus with a third reviewer (KKT). Titles, abstracts, and full-text articles were included if they discussed cases of surgical management of RIBPN in patients who received radiation for breast cancer treatment. Letters to the editor and articles not in English were excluded. Articles examining the epidemiology, pathogenesis, and clinical presentation of RIBPN or non-surgical treatment of RIBPN were collected, however, they were only included in the analysis if they contained primary data. References from included articles were examined to ensure that no relevant citations were overlooked. 306 studies were compiled with nine articles meeting inclusion criteria. 297 articles were excluded, including 144 duplicates and 153 non-pertinent articles (Fig. 1).

**Fig. 2:**
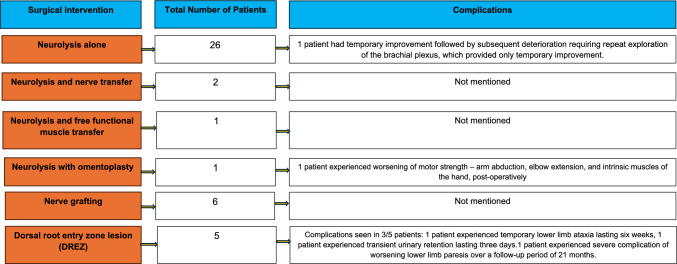
Complications flow chart

Patient pain was assessed using the Visual Analogue Scale (VAS), and motor strength was evaluated via physical exams and motor unit potentials [13]. Muscle strength and recovery were gauged using the Medical Research Council (MRC) grading system. The LENT-SOMA index was used to evaluate pain, sensation, and motor strength. These tools helped evaluate the effectiveness of surgical treatments for RIBPN (Fig. 2).

Risk of bias within each included study was systematically assessed using the ROBINS-I (Risk Of Bias In Non-randomized Studies of Interventions) tool, evaluating seven domains: confounding, selection of participants, classification of interventions, deviations from intended interventions, missing data, measurement of outcomes, and selective reporting. Each domain was independently evaluated assigning bias levels as low, moderate, serious, or critical. The overall risk of bias for each study was determined by the highest level of bias identified within any single domain. Detailed results of the risk of bias assessment are presented in the supplementary ROBINS-I Risk of Bias Assessment Table.

**Fig. 3:**
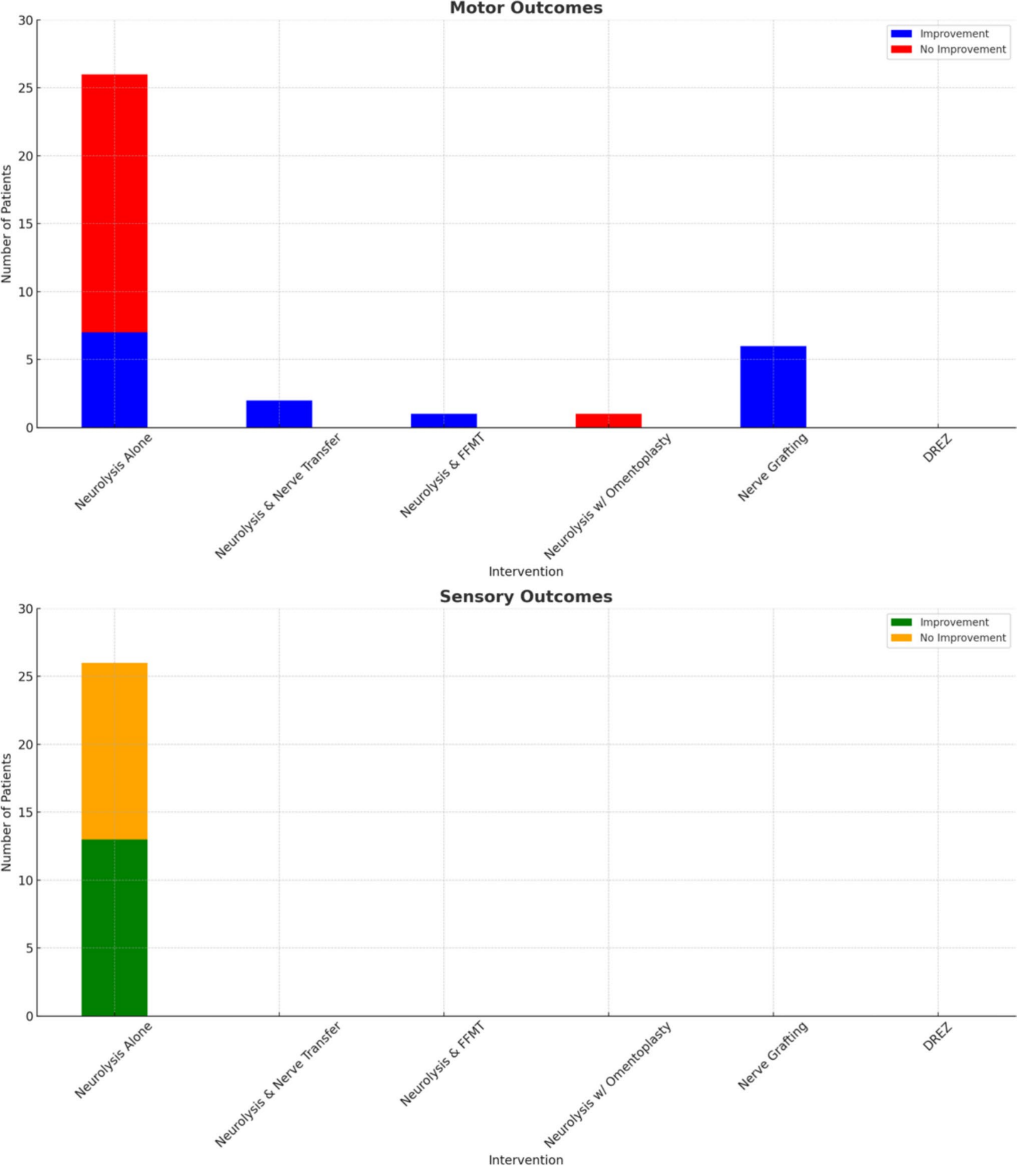

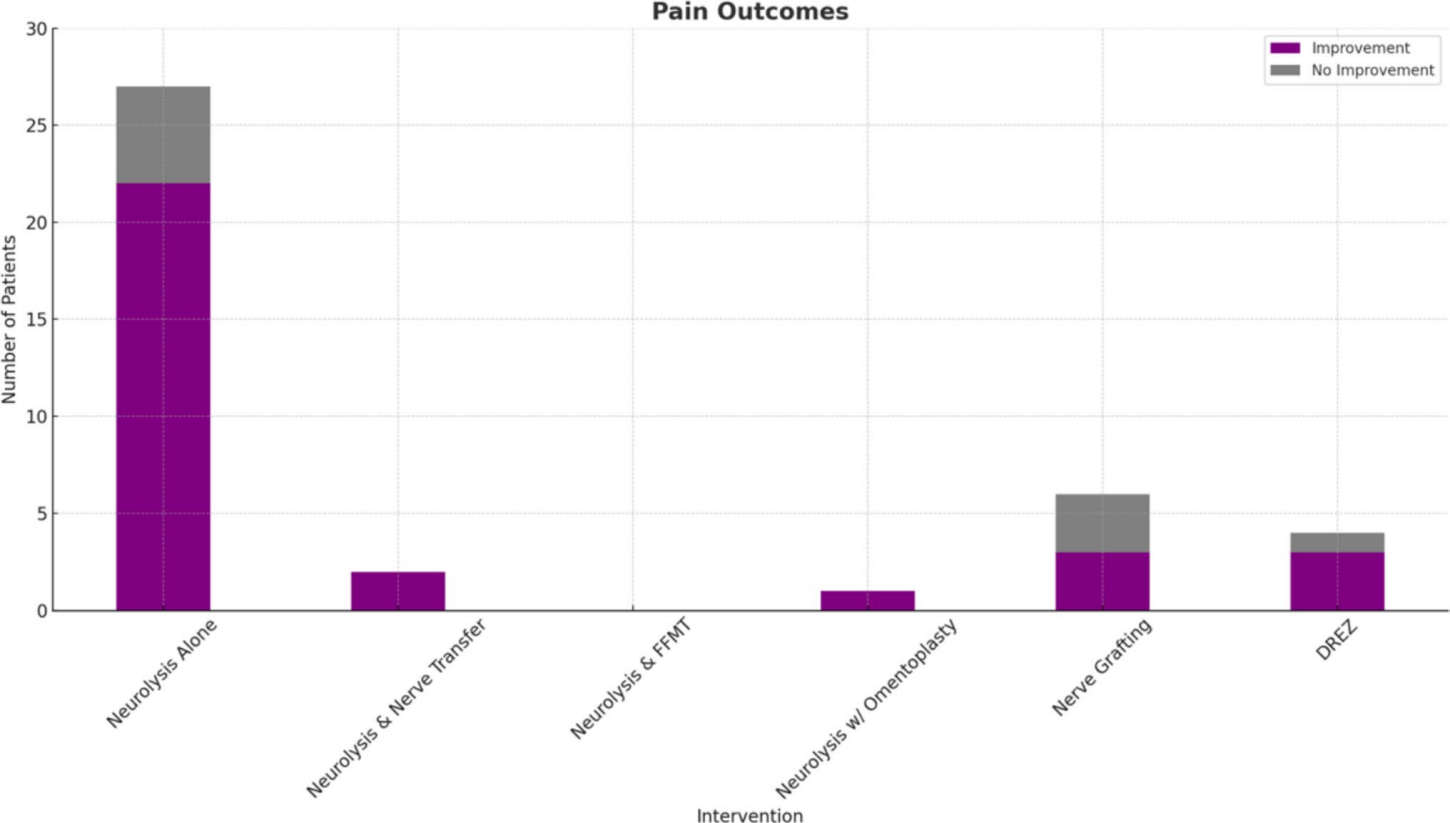
Outcomes analysis

The protocol for this systematic review was prospectively registered with PROSPERO (International Prospective Register of Systematic Reviews) under registration number 1113294. It was developed in accordance with PRISMA-P (Preferred Reporting Items for Systematic Review and Meta-Analysis Protocols) guidelines and includes predefined objectives, eligibility criteria, and methods for data collection and synthesis (Fig. 3).

## Results

Out of 306 eligible studies, nine met the inclusion criteria. A total of 41 breast cancer patients underwent surgery for RIBPN; four cases were reported in case studies.

### Demographics

Demographic data and treatment characteristics are summarized in Tables 1, 2 and 3. Eight of studies included only female patients; one study did not specify patient gender. The mean age was 55, with a range of 29–70 years. Six studies included only patients with RIBPN related to breast cancer. Three studies included patients treated for other cancers, such as nasopharyngeal cancer, desmoid tumors and parotid gland neoplasms, although non-breast cancer cases were omitted from the final analysis [14, 15]. Thus, all 41 patients included in the analysis received adjuvant radiation for breast cancer. Details regarding radiation history were provided for some patients (Tables 2 and 3).

**Table 1:** Summary of included studies

Source	Title	Reference	Study sample	Surgical treatment	Results
PubMed	Outcome of Nerve Grafting for Radiation-Induced Brachial Plexopathy	Yaobin Yin et al [12]	6	Segmental nerve resection and autografting: musculocutaneous nerve reconstruction with sural nerve grafting	Return of antigravity elbow flexion, mean VAS for pain improved from 2.2 preoperatively to 0.7 postoperatively
PubMed	Radiation-induced brachial plexus neuropathy: A review	Anshu C Warade et al [5]	11	External neurolysis of the supra-and infraclavicular brachial plexus. Perineural scar tissue excision	-9 patients had significant improvement in neurogenic pain and paresthesia -2 patients had no significant relief in sensory symptoms and pain persisted -Motor weakness improved proximally at shoulder and arm in 3 patients -For 9 patients, distal motor weakness failed to improve and deteriorated significantly over a 6-months
PubMed	Treatment of radiation-induced brachial plexopathy with omentoplasty	Adilson José Manuel de Oliveira et al [3]	1	Extensive external microneurolysis and omentoplasty	Improvement in symptoms: At 6-month post-op, tingling in the lateral palmar region and dorsal region of the hand, and the lateral areas of the upper arm and forearm remained, lymphedema improved, VAS for pain improved to 0/10 from 10/10, motor strength was equivalent to preop strength in arm adduction (grade 3) and elbow flexion (grade 0); it was worse (grade 2) in arm abduction, elbow extension, and the intrinsic muscles of the hand
PubMed	Nerve Transfer for Elbow Flexion in Radiation-Induced Brachial Plexopathy: A Case Report	TH Tung et al[11]	1	Double fascicular transfer of median and ulnar nerves with internal neurolysis	Improvement in symptoms: At 6-month post-op, patient had reinnervation of elbow flexors with detectable voluntary muscle contraction. At 2 years, patient improved to MRC grade 4/5 elbow flexion strength. Physical examination of hand function showed FDS function to all digits and FCU wrist function, no functional donor morbidity noted
PubMed	Free Functioning Muscle Transfer in Radiation-Induced Brachial Plexopathy: Case Report	Bipin A. Gangurde et al [8]	1	Gracilis free muscle transfer and neurolysis of the infraclavicular region around the lateral cord and MCN bifurcation	Improvement in symptoms: 2yrs after FFMT, amplitude of motor unit potentials increased to 4 mV and patient was able to flex elbow from 40-degree to 110-degree with full finger extension in 10-degree wrist flexion and was able to flex the fingers. Able to voluntarily grasp and hold small objects and perform most activities of daily living, such as holding down while cutting with a knife held in the contralateral hand. The shoulder active range of motion unchanged. The Disabilities of Arm, Shoulder, and Hand functional score improved from 56 preoperatively to 21. No progression of neurological symptoms occurred
PubMed	Dorsal root entry zone lesions for treatment of pain-related to radiation-induced plexopathy	Manoel Jacobsen Teixeira et al [14]	5	Cervical thoracic laminectomy, microsurgical dissection of the dorsal root entry zone followed by dorsal root entry zone lesions	Three patients experienced complete relief of symptoms, one patient experienced moderate relief and one patient experienced complete relief after additional lesion
EMBASE	Radiation-Induced Brachial Plexopathy in Breast Cancer and the Role of Surgical Treatment	Kenan Kibici et al [41]	11	External neurolysis and decompression	No significant improvement was observed in sensory or motor functions, mean VAS decreased from 9.4 preoperatively to 4 postoperatively
EMBASE	Partial Ulnar Nerve Transfer to the Nerve to the Biceps for the Treatment of Brachial Plexopathy in Metastatic Breast Carcinoma: Case Report	Manzhi Wong et al [16]	1	Full brachial plexus exploration, neurolysis, and transfer of 1 ulnar nerve fascicle to the nerve to the biceps brachii	Patient experienced improved neuropathic pain, improved elbow flexion against gravity, biceps power against resistance and Tinel sign elicited at level of biceps, persistent C6-C7 dermatomal numbness, good function of hand intrinsic muscles, successful reinnervation of the right biceps muscle based on nerve conduction studies 2 years post-op, good ulnar innervation of bicep. NCS at 2 years post-op confirmed successful reinnervation of the right biceps muscle
OTHER	Brachial plexus injuries after radiotherapy—analysis of 6 cases	Jerzy Gosk et al [15]	4	3 patients underwent neurolysis, 1 patient underwent double neurolysis	After surgery, one patient experienced a significant improvement in pain with decreased pain and sensory disorder and decreased motor deficits. Another patient experienced temporary improvement in pain and sensory deficits over a 2-year period, after which the patient’s status deteriorated. The patient underwent repeat exploration. Two patients did not have significant improvement in their symptoms after surgical management

**Table 2:** Full summary of reviewed studies

Source	Title	Authors	# of patients	Mean age (years)	Presentation	Medical Treatment	Chemotherapy	Radiation therapy
PubMed: PMID 36519879	Outcome of Nerve Grafting for Radiation-Induced Brachial Plexopathy	Yaobin Yin et al [12]	6	52 (range 38–64)	- numbness or mild pain of affected limb - gradual loss of muscle strength in upper limb - elbow flexion dysfunction - MRC grade 2 or less	Unknown	Unknown	Yes
PubMed: PMID 30688233	Radiation-induced brachial plexus neuropathy: A review	Anshu C Warade et al [5]	11	48 (range 42–65)	- severe neurogenic pain (VAS 9–10) and severe paresthesia - pan-brachial plexus neuropathy - weakness of all muscle groups of upper limb - lymphedema in 7 patients at time of surgery	Oral analgesics and pregabalin for minimum of 3–4 months	Yes	Yes: adjuvant local radiotherapy (50–55 Gy)
PubMed: PMID 33344306	Treatment of radiation-induced brachial plexopathy with omentoplasty	Adilson José Manuel de Oliveira et al [3]	1	68	- tingling in hand, hypoesthesia, and loss of strength in upper limb - neuropathic pain in upper limb (VAS 10/10) mainly in hand and lymphedema - motor strength grade 4 for arm abduction; 3 for arm adduction, elbow extension, and intrinsic muscles of the hand; and 0 for elbow flexion. LENT–SOMA grade 4	pregabalin, amitriptyline, dipyrone, and venlafaxine	Yes	Yes
PubMed: PMID 18843522	Nerve Transfer for Elbow Flexion in Radiation-Induced Brachial Plexopathy: A Case Report	Thomas H. Tung et al [11]	1	59	Marked neurologic dysfunction in the RUE -constant tingling and numbness in her R arm and the median nerve distribution of the R hand and palm -no active elbow flexion -little movement of shoulder -frequently dropping “things” w her R hand -muscle cramping and increased pain with activity -R shoulder, neck and rib pain, pain 3–4/10 -swelling in R arm but not hand	hyperbaric treatment for delayed wound healing -unknown other medical management	Yes: combination chemo	Yes: 48 Gy of external beam radiation divided into 38 treatments over 4 weeks, hyperthermia, and radium bead implants to the right chest wall and axilla
PubMed: PMID 25155695	Free Functioning Muscle Transfer in Radiation-Induced Brachial Plexopathy: Case Report	Bipin A. Gangurde et al [8]	1	56	- neurologic dysfunction of left upper extremity, loss of shoulder movements, elbow flexion, weak wrist, and finger extension	Unknown	Yes	Yes: external beam radiation of 200 Gy, divided into 30 treatments over 6 weeks to the left chest wall and axilla followed by 200 Gy of radiation in 25 divided doses over 4 weeks to the supraclavicular region
PubMed: PMID 17471080	Dorsal root entry zone lesions for treatment of pain-related to radiation-induced plexopathy	Manoel Jacobsen Teixeira et al [14]	5	48.4	edema, muscle atrophy, cutaneous dystrophy, and pain, motor deficits: 4 patients with monoplegia of affected limb, 1 patient with monoparesis and posterior cord syndrome of lower limb on affected side, sensory deficits: 3 patients with anesthesia in C5 – T1, 1 patient with hypoesthesia in C5 – T1, 1 patient with hypoesthesia/hyperpathia in C4 – T4	NSAIDs, Carbamazepine, Tricyclic antidepressants, Neuroleptics, Opioids	Unknown	Yes
EMBASE	Radiation-Induced Brachial Plexopathy in Breast Cancer and the Role of Surgical Treatment	Kenan Kibici et al	11	42.54	- severe pain, weakness, and loss of sensation in the affected shoulder and arm - hypoesthesia on the affected arm and all reflexes were abolic - active movement in the affected shoulder was 5 to 10-degrees in all directions, shoulder flexion and abduction was one-fifth, elbow flexion and extension was one to two-thirds, wrist flexion and extension was one to two-fifths, and flexion and extension of the fingers was one to two fifths -trophic changes were present in the affected extremity -all patients had LENT-SOMA grade 3 or 4 -mean VAS 9.4	Gabapentin, pregabalin, amitriptyline, tramadol HCl, pethidine HCl	Yes (9/11)	Yes
EMBASE	Partial Ulnar Nerve Transfer to the Nerve to the Biceps for the Treatment of Brachial Plexopathy in Metastatic Breast Carcinoma: Case Report	Manzhi Wong et al [16]	1	65	-R upper limb pain, constant and severe pain (pain score 9/10) radiating from the shoulder distally, associated with right thumb and index finger numbness, R shoulder and elbow weakness - MRC grade 0 in supraspinatus, infraspinatus, deltoid, coracobrachialis, pectoralis major, biceps brachii, and brachialis muscles. Wrist and finger extension and flexion were graded M4. She had hypoesthesia in the C6 and C7 dermatome distribution. A Tinel’s sign was elicited in the supraclavicular fossa	tramadol, gabapentin, amitriptyline, and ketamine infusion	Yes	Yes: external beam radiotherapy to the supraclavicular and axillary regions, with a total dose of 6000 cGy delivered in 30 fractions over a 6-week period
Cited article	Brachial plexus injuries after radiotherapy—analysis of 6 cases	Jerzy Gosk et al [15]	4	56 (40–64)	-pain, sensory and motor deficits -LENT-SOMA Grade 4 in all 4 patients	Unknown	Yes (2/4 patients underwent chemotherapy)	Yes: -1 patient 50 Gy/50 Gy -1 patient 45 Gy/45 Gy -1 patient 45 Gy -1 patient unknown

Source	Surgical treatment of RIBPN	Mean follow up duration post-surgery	Number of patients with improvement in symptoms	Avg of time from radiation to onset of symptoms	Distal symptoms in hand?	EMG/Nerve conduction studies	Imaging studies to aid in diagnosis	Time from symptom onset to surgical treatment
PubMed: PMID 36519879	Segmental nerve resection and autografting: musculocutaneous nerve reconstruction with sural nerve grafting	Follow up every three months with avg total follow up time of 32.3 months (17 – 72 months)	Muscle strength, based on muscle strength in biceps, improved for all 6 patients. Mean muscle strength of biceps improved from M = 0.17 preoperatively to M = 3.5 post-operatively. 5 patients experienced improvement in active range of motion with movement of elbow extension to flexion. 1 patient experienced improvement in active range of motion with movement of elbow extension to flexion and shoulder adduction to abduction. Pain improved in 3/6 patients. Mean VAS improved from 2.7 preoperatively to 0.7 post-operatively. 2/6 patients were not experiencing pain prior to surgery and thus experienced no Change in VAS. 1 patient did not experience improvement in pain	9.45 years (range 0.7–21 years)	Yes: weakness in hand intrinsic muscles, finger extension and flexion	Yes	MR neuroimaging (MRN)	21.2 months (7 months–48 months)
PubMed: PMID 30688233	External neurolysis of the supra- and infraclavicular brachial plexus. Perineural scar tissue excision	11 months (6–22 months)	-9 patients had significant improvement in neurogenic pain and paresthesia -2 patients had no significant relief in sensory symptoms and pain persisted - Motor weakness improved proximally at shoulder and arm in 3 patients -For 9 patients, distal motor weakness failed to improve and deteriorated significantly over a 6-month period	18.7 months	Yes: distal muscle weakness involving the grip severely affected (more than proximal muscle weakness)	Yes: all patients underwent EMG-NC	Yes: all patients underwent preoperative MRI, which did not differentiate and distinguish reoccurrence of tumor v fibrosis - FDG-PET CT did not demonstrate any increase in metabolism involving the brachial plexus and metastasis elsewhere	Unknown
PubMed: PMID 33344306	Extensive external microneurolysis and omentoplasty	Total time not specified	At 6 month post-op, tingling in the lateral palmar region and dorsal region of the hand, and the lateral areas of the upper arm and forearm remained, lymphedema improved, VAS for pain improved to 0/10, motor strength was equivalent to preop strength in arm adduction (grade 3) and elbow flexion (grade 0); it was worse (grade 2) in arm abduction, elbow extension, and the intrinsic muscles of the hand. LENT–SOMA grade 2	18 years	Yes: tingling in R hand (tingling in lateral palmar region, dorsal region of hand and lateral areas of upper arm and forearm) and hypoesthesia in the R upper limb. Decreased motor strength (graded 3 for intrinsic hand muscles)	Unknown	Unknown	1 year
PubMed: PMID 18843522	Double fascicular transfer of median and ulnar nerves with internal neurolysis	24 months	-at 6mo post-op, reinnervation of elbow flexors with detectable voluntary muscle contraction -at 2 years, improved to MRC grade 4/5 elbow flexion strength. Physical examination of hand function showed FDS function to all digits and FCU wrist function. No functional donor morbidity noted	Progressive over several years	Yes: frequently dropping things, however on physical exam hand function was intact, pressure over the median nerve in the right proximal forearm produced increased paresthesia in the median nerve distribution of the right hand	Yes: good function in the supra- and infraspinatus muscles but absent responses in R biceps or deltoid, lateral antebrachial cutaneous nerve, and a right axillary response was unobtainable. EMG demonstrated isolated small motor units recordable from the deltoid, biceps, and brachioradialis at rest	PET scan to rule out neoplastic process	Not specified
PubMed: PMID 25155695	Gracilis free muscle transfer and neurolysis of the infraclavicular region around the lateral cord and MCN bifurcation	24 months	2yrs after FFMT, amplitude of motor unit potentials increased to 4 mV and patient able to flex elbow from 40 to 110 degree with full finger extension in 10-degree wrist flexion and was able to flex the fingers. Able to voluntarily grasp and hold small objects and perform most activities of daily living, such as holding down while cutting with a knife held in the contralateral hand. The shoulder active range of motion unchanged The Disabilities of Arm, Shoulder, and Hand functional score improved from 56 preoperatively to 21. No progression of neurological symptoms	7 years	Yes: -weak wrist and finger extension - full finger flexion but weak extension (BMRC grade 2) - grip strength was 1 kg compared with 32 kg on the other side - hypoesthesia in C7 - able to perceive the 2.4-g Semmes–Weinstein monofilament but not the 0.22-g monofilament on the ulnar 3 fingers. The radial artery was faintly palpable at the wrist	Yes: EMG findings supported a diagnosis of RIBP	Yes: MRI findings supported diagnosis of RIBP	Unknown
PubMed: PMID 17471080	Cervical thoracic laminectomy, microsurgical dissection of the dorsal root entry zone followed by dorsal root entry zone lesions	14.4 months	5: three patients experienced complete relief of symptoms, one patient experienced moderate relief and one patient experienced complete relief after additional lesion	Unknown	Unknown	Yes: EMG findings showing severe denervation	Yes: CT or MRI to r/o neoplastic causes of neuropathy	Unknown
EMBASE	External neurolysis and decompression	6 months	0: Mean VAS value decreased from 9.4 to 4. No significant improvement was observed in sensory or motor functions	12.36 months	Yes: hand weakness	Yes	Yes: MRI	Unknown
EMBASE	full brachial plexus exploration, neurolysis, and transfer of 1 ulnar nerve fascicle to the nerve to the biceps brachii	24 months	Improvement in neuropathic pain, improved elbow flexion against gravity, biceps power against resistance and Tinel sign elicited at level of biceps, persistent C6–C7 dermatomal numbness, good function of hand intrinsic muscles, successful reinnervation of the right biceps muscle based on nerve conduction studies 2 years post-op, good ulnar innervation of bicep	N/A	Yes: right thumb and index finger numbness, wrist and finger extension and flexion were graded M4	Yes: EMG showed upper trunk plexopathy, spontaneous fasciculations in the infraspinatus, muscle, no denervation in the mid- cervical paraspinal muscles	Yes: MRI showed diffuse swelling and enhancement of the R brachial plexus extending from the roots to the cords, inflammation, and edema of the adjacent soft tissues in the axilla and right upper chest wall, and the changes were attributed to radiotherapy	13 months
Cited article	3 patients underwent neurolysis, 1 patient underwent double neurolysis	Unknown	2 patients experienced improvement in sensory, motor and pain symptoms. One patient experienced transient improvement in pain and sensory symptoms with subsequent deterioration	7 years (1–20 years)	Unknown	Unknown	Unknown	25.6 years (2.5–46 years)

**Table 3:** Surgical treatments and associated outcomes

Intervention	Total number of patients	Demographic data	Nuances	Outcomes
Neurolysis alone	26	- All patients were female - Age: 46.92 years (40–65) - Presentation: severe neurogenic pain, paresthesia, pan-brachial plexus neuropathy, weakness of muscle groups in affected limb, trophic changes, decreased active movement - Medical treatment: 11 patients had oral analgesics and pregabalin for minimum of 3–4 months. 11 patients had been managed with gabapentin, pregabalin, amitriptyline, tramadol HCl and/or pethidine HCl. For 4 patients, prior medical treatment not specified - Chemotherapy: 22 patients underwent prior chemotherapy. 4 patients did not undergo chemotherapy - Radiation: All 26 patients underwent prior radiation therapy - Avg time from radiation onset to start of symptoms: 15.53 months *does not include 4 patients from Gosk et al - Distal symptoms in hand: 11 patients had distal muscle weakness primarily involving the grip, which was severely affected. 11 patients had significant hand weakness, including weakness in finger flexion and extension. For 4 patients, distal hand symptoms were not provided - EMG/nerve studies: at least 22/26 patients had prior EMG-NC study of brachial plexus, revealing severe brachial plexopathy and denervation changes in the brachial plexus - Imaging to aid in dx: at least 22/26 patients underwent MRI prior to surgery - Time from symptom onset to surgical management: Not specified for 22/26 cases. However, for 4 cases, it was 25.6 years (2.5 – 46 years) - Mean follow up time: 8.5 months *does not include 4 patients from Gosk et al	-All patients underwent external neurolysis -17 patients underwent neurolysis of supraclavicular and infraclavicular brachial plexus. 5 patients underwent neurolysis of infraclavicular region only. 2 patients underwent only supraclavicular incision, while 2 patients underwent both supraclavicular and axillary incisions -In all cases, excised tissue was sent for histopathologic analysis and confirmed fibrous and scar tissue -At least 11 patients had biopsies of excised tissue, which failed to reveal tumor in all 11 cases	Motor: 1 patient experienced significant reduction in motor deficits following neurolysis. This patient underwent both supraclavicular and axillary incisions. 3 patients had a mild improvement in motor strength from M0 preoperatively to M1 post-operatively. These 3 patients had infraclavicular incisions only. Finally, 3 patients had motor weakness improve proximally at the shoulder and arm. These 3 patients underwent both supraclavicular and infraclavicular neurolysis. 19 patients had no improvement in motor strength, and motor deficits persisted post-operatively. Of these patients, 13 patients underwent both supraclavicular and infraclavicular incisions, 3 underwent infraclavicular only, 2 underwent supraclavicular incisions only, 1 underwent supraclavicular and axillary incisions Sensory: 10 patients had significant improvement in sensory symptoms. Of these patients, 9 underwent supraclavicular and infraclavicular incisions and 1 underwent supraclavicular and axillary incisions. 3 patients had a mild improvement in sensory deficits from S0 preoperatively to S1 post-operatively. 2 of these patients underwent infraclavicular incisions only and 1 patient underwent both supraclavicular and infraclavicular lesions. 1 patient experienced temporary improvement in sensory symptoms followed by subsequent deterioration and 12 patients had no improvement in sensory symptoms Pain: 21 patients experienced a significant improvement in pain, with pain reduction. 1 patient had a transient improvement in pain followed by subsequent deterioration. 4 patients had persistence in pain
Neurolysis and nerve transfer	2	- All patients were female **- **Mean age: 62 - Presentation: severe pain (9/10) radiating from the shoulder distally, thumb and index finger numbness, shoulder pain, elbow weakness, spontaneous fasciculations, inflammation and edema, tingling and numbness in arm and median nerve distribution of hand and palm, loss of elbow flexion and little movement of shoulder, dropping things from hand, muscle cramping - Medical treatment: Yes, tramadol, gabapentin, amitriptyline, ketamine, hyperbaric treatment - Chemotherapy: Yes - Radiation: Yes - Avg time from radiation onset to start of symptoms: Not specified - Distal symptoms in hand: Yes, thumb and index finger numbness, - EMG/nerve studies: Yes - Imaging to aid in dx: Yes, one patient underwent MRI and one patient underwent PET - Time from symptom onset to surgical management: One patient 13 months, one patient unknown - Mean follow up time: 24 months	-1 patient (Tung et al. study) underwent neurolysis and scar tissue release. Next, the patient underwent nerve transfer reconstruction with double fascicular transfer technique using the median and ulnar nerves as donors. Internal neurolysis and fascicular dissection was performed to dissect individual motor fascicles. No functional donor morbidity was noted: patient maintained good hand fx, sensation and strength - 1 patient (Wong et al. study) underwent full brachial plexus exploration, neurolysis, and transfer of 1 ulnar nerve fascicle to the nerve to the biceps brachii. Tissue histology of the right brachial plexus nerve sheath showed infiltrative carcinoma involving the nerve bundles and right anterior chest wall mass was positive for cancer cells confirmed to be metastatic deposits of ductal adenocarcinoma. Patient was given radiotherapy and continued on tamoxifen, and she has since remained in clinical remission. No ulnar nerve deficit. normal 2-point discrimination in the small finger (4 mm) post-operatively	Motor: 2/2 patients experienced improvement in motor function. For one patient, after 6 months, she had gravity-eliminated elbow flexion (M2) and improving finger flexion. She had elbow flexion against gravity (M3) at 1 year. At the 18-month follow-up, biceps power was M4 (against resistance) and Tinel’s sign was elicited at the level of the biceps. There was active abduction of her shoulder joint to 45° (supraspinatus and deltoid power M3) and good function of the hand intrinsic muscles. For the second patient, after six months, patient had reinnervation of her elbow flexors with detectable voluntary muscle contraction. At 2 years, she had recovered MRC grade 4/5 elbow flexion strength. Physical examination of hand function demonstrated intact FDS function to all digits and FCU wrist function, illustrative of the redundancy of innervation to these structures Sensory: Not described in detail, however for both patients, donor sensation was maintained post-operatively. One patient had persistent C6–C7 dermatomal numbness post-operatively Pain: Both patients experienced a significant improvement in pain post-operatively with full resolution of neuropathic pain seen in on patient
Neurolysis and free functional muscle transfer	1	- All patients were female - Age: 56 years - Presentation: Loss of shoulder movements, elbow flexion, weak wrist and finger extension. Elbow flexion grade 0 and extension was grade 3. Active shoulder abduction, flexion, external and internal rotation were 20degrees, 20degrees, 60degrees, and 70degrees, respectively. Passive shoulder abduction was 100 degrees. She was able to actively extend the wrist to neutral position (BMRC grade 2). The serratus anterior was working because its power was BMRC grade 3. The C5-C6 dermatomes were anesthetic and there was hypo- esthesia in the C7 area. She was able to perceive the 2.4-g Semmes–Weinstein monofilament but not the 0.22-g monofilament on the ulnar 3 fingers. The radial artery was faintly palpable at the wrist - Medical treatment: Not specified - Chemotherapy: Yes - Radiation: external beam radiation of 200 Gy, divided into 30 treatments over 6 weeks to the left chest wall and axilla followed by 200 Gy of radiation in 25 divided doses over 4 weeks to the supraclavicular region - Avg time from radiation onset to start of symptoms: 7 years - Distal symptoms in hand: Full finger flexion but weak extension (BMRC grade 2). Left hand grip strength was 1 kg compared with 32 kg on the right side - EMG/nerve studies: Yes, supported diagnosis of RIBPN - Imaging to aid in dx: Yes, MRI findings supported diagnosis of RIBPN - Time from symptom onset to surgical management: unknown - Mean follow up time: 24 months	-Spinal accessory nerve was selected as donor nerve -Gracilis muscle was selected as donor muscle -Patient underwent surgery for reconstruction of finger extension and elbow flexion -Spinal accessory nerve was found on anterior surface of trapezius and its terminal branch as dissected - Neurolysis was performed in the infraclavicular region around the lateral cord and MCN bifurcation	Motor: The patient experienced an improvement in motor symptoms, specifically in elbow flexion and finger extension. 2yrs after FFMT, amplitude of motor unit potentials increased to 4 mV and patient able to flex elbow from 40-degree to 110-degree with full finger extension in 10-degree wrist flexion and was able to flex the fingers. Able to voluntarily grasp and hold small objects and perform activities of daily living, such as holding down while cutting with a knife held in the contralateral hand. The shoulder active range of motion unchanged. The Disabilities of Arm, Shoulder, and Hand functional score improved from 56 preoperatively to 21 Sensory: Not specified Pain: Not specified
Neurolysis with omentoplasty	1	**-** The patient was female - Age: 68 years - Presentation: Tingling in the right hand in the lateral palmar region and the dorsal region, progressive hypoesthesia in the right upper limb, progressive loss of strength in the right upper limb with motor strength graded 4 for arm abduction, 3 for arm adduction, elbow extension and intrinsic muscles of hand and 0 for elbow flexion. LENT-SOMA grade 4. Neuropathic pain (VAS 10/10) in the upper limb (mainly in hand), and lymphedema - Medical treatment: pregabalin, amitriptyline, dipyrone, and venlafaxine - Chemotherapy: Yes - Radiation: Yes - Avg time from radiation onset to start of symptoms: 18 years - Distal symptoms in hand: Yes, tingling in R hand (tingling in lateral palmar region, dorsal region of hand), decreased motor strength (graded 3 for intrinsic hand muscles) - EMG/nerve studies: Not specified - Imaging to aid in dx: Not specified - Time from symptom onset to surgical management: 1 year - Mean follow up time: Not specified	-Supraclavicular and infraclavicular brachial plexus exploration -Main intraoperative findings were fibrotic tissue adherent to the supraclavicular brachial plexus and a neuroma in continuity in lateral cord -Extensive external microneurolysis was performed and a graft of omentum was harvested through a median supra umbilical laparotomy. Vascular pedicle was anastomosed to cervical transverse vessels and graft was placed over the entire brachial plexus	Motor: The patient experienced worsening motor strength. The patient’s motor strength was equivalent to her preoperative strength in arm adduction (grade 3) and elbow flexion (grade 0); however, it had become worse (grade 2) in arm abduction, elbow extension, and the intrinsic muscles of the hand. LENT-SOMA grade 2 Sensory: Sensory function remained relatively unchanged. At the 6-month follow-up, she was still experiencing tingling in the lateral palmar region, the dorsal region of the hand, and the lateral areas of the upper arm and forearm Pain: Patient experienced a significant improvement in pain, with neuropathic pain achieving VAS 0/10 and driving a progressive pain killer withdrawal. Additionally, lymphedema improved
Nerve grafting	6	**-** All patients were female - Age: 52 years (38 – 64 years) - Presentation: - Medical treatment: Not specified - Chemotherapy: Not specified - Radiation: Yes - Avg time from radiation onset to start of symptoms: 9.45 years (0.7 – 21 years) - Distal symptoms in hand: Yes, weakness in hand intrinsic muscles, finger extension and flexion - EMG/nerve studies: Yes - Imaging to aid in dx: Yes, MRN - Time from symptom onset to surgical management: 21.2 months (7 – 48 months) - Mean follow up time: 32.3 months (17 – 72 months)	-2/6 patients had undergone neurolysis with no improvement in symptoms	Motor: Muscle strength, based on muscle strength in biceps, improved for all 6 patients. Mean muscle strength of biceps improved from M = 0.17 preoperatively to M = 3.5 post-operatively. 5 patients experienced improvement in active range of motion with movement of elbow extension to flexion. 1 patient experienced improvement in active range of motion with movement of elbow extension to flexions and shoulder adduction to abduction Sensory: Details not provided Pain: Pain improved in 3/6 patients. Mean VAS improved from 2.7 preoperatively to 0.7 post-operatively. 2/6 patients were not experiencing pain prior to surgery and thus experienced no Change in VAS. 1 patient did not experience improvement in pain
Dorsal root entry zone lesion (DREZ)	5	- All patients were female - Age: 48.4 years - Presentation: pain (mean preop VAS = 9.2), hypoesthesia or anesthesia in affected limb, hyperpathia, monoplegia in affected limb, monoparesis of affected limb and posterior cord syndrome of lower limb on affected side - Medical treatment: NSAIDs, carbamazepine, tricyclic antidepressants, neuroleptics. 2 patients used opioids for pain - Chemotherapy: Not specified - Radiation: Yes - Avg time from radiation onset to start of symptoms: Not specified - Distal symptoms in hand: Not specified - EMG/nerve studies: Yes, EMG findings showing severe denervation - Imaging to aid in dx: Yes, CT or MRI to r/o neoplastic causes of neuropathy - Time from symptom onset to surgical management: Not specified - Mean follow up time: 14.4 months	-1/5 patients had prior microneurolysis × 3 for RIBPN, which no/little improvement. 2 patients had no prior surgical management. 1 patient had prior anesthetic blocks. 1 patient had prior anesthetic blocks and intercostal rhizotomy spinal cord stimulation -2 patients experienced no adverse effects. 1 patient experienced temporary lower limb ataxia lasting 6 weeks. 1 patient experienced transient urinary retention lasting 3 days. 1 patient experienced worsening lower limb paresis - Results were categorized based on pain symptoms using VAS - Dorsal root entry zone lesions were performed following cervical thoracic laminectomy and microsurgical dissection of the dorsal root entry zone ipsilateral to the side of pain - All patients experienced transient nociceptive pain at the incision site immediately after surgery, which was adequately controlled with analgesics	Motor: Not described Sensory: Not described Pain: 3/5 patients experienced complete relief of pain symptoms immediately after DREZ, which persisted throughout follow up period. 1/5 patients experienced moderate relief in symptoms and an additional lesion was performed, which led to complete relief of symptoms. 1/5 patients experienced moderate relief in symptoms

### Presentation and Medical Management

The timing of radiation exposure to symptom onset varied considerably among the subjects included in the analysis. On average, symptoms emerged 48.8 months following radiation, with a range spanning from 8.4 to 252.2 months. The most common symptoms among all study patients were neurogenic pain, paresthesia, motor weakness, sensory loss, and pan-brachial plexus neuropathy. All 41 patients (100%) reported muscle weakness. Additionally, 26 patients (63.4%) had symptoms affecting hand function, such as reduced motor strength in the intrinsic hand muscles, muscles of finger flexion and extension, and wrist flexion and extension. Other hand symptoms included numbness and tingling in one patient (2.4%), decreased grip strength in two patients (4.9%), and instances of dropping objects and localized numbness in another patient (2.4%) [5, 8, 11, 16].

Medical treatments for RIBPN included oral analgesics, pregabalin, amitriptyline, dipyrone, venlafaxine, tramadol, carbamazepine, neuroleptics, opioids and pethidine. The indication for surgery was failure to respond to medical management in six studies. In two studies, surgery was performed based on symptom severity (LENT-SOMA grade 3 or 4) or progression of disease. Average time from radiation to surgery was not well quantified. All 41 patients were analyzed further for surgical details.

### Surgical Treatment Categories

Six surgical treatments were performed for RIBPN. External neurolysis alone was the most common surgical treatment, used in three studies (26/41 patients; 61.9%). Of these patients, 11 (26.8%) underwent external neurolysis of the supra- and infraclavicular brachial plexus with perineural scar tissue excision. Another 11 (26.8%) patients underwent decompression and external neurolysis of the affected middle and/or lower truncus nerves. For the remaining four patients (9.8%) who underwent external neurolysis, the exact nerves were not specified. The second most performed procedure was segmental nerve resection and autograft reconstruction with sural nerve grafting to the musculocutaneous nerve (6/41; 14.6%). Five patients (5/41; 12.2%) underwent cervical thoracic laminectomy with microsurgical dissection and dorsal root entry zone lesioning. Two patients (2/41; 4.9%) underwent neurolysis with nerve transfer. One of these patients (1/41; 2.4%) underwent full brachial plexus exploration, neurolysis, and transfer of one ulnar nerve fascicle to the biceps brachii. The other patient (1/41; 2.4%) underwent double fascicular transfer of median and ulnar nerves with internal neurolysis. One patient (1/41; 2.4%) underwent neurolysis and omentoplasty whereby an omental graft was harvested with a median supra umbilical laparotomy and a vascular pedicle was anastomosed to cervical transverse vessels and placed over the brachial plexus (1/41; 2.4%) [3]. Finally, one case report described a patient who underwent gracilis-free muscle transfer and neurolysis at the infraclavicular region around the lateral cord and the musculocutaneous nerve bifurcation (1/41; 2.4%) [8].

### Surgical Treatment Outcomes

The average duration of follow-up post-surgery was 19.4 months. For 11 patients undergoing external neurolysis and perineural scar tissue excision, three (33.3%) experienced proximal motor improvement at the shoulder and arm; however nine patients (81.8%) experienced worsening distal motor weakness over six months [5]. Nine patients (81.8%) experienced significant improvement in neurogenic pain and paresthesia while two patients (18.2%) did not [5]. Additionally, in the study by Kibici et al., involving 11 patients treated with decompression and external neurolysis, only three patients (27.3%) reported mild improvement in motor and/or sensory symptoms. However, all 11 patients (100%) experienced pain reduction, with VAS (Visual Analogue Scale) scores dropping from 9.4 to 4. Average follow-up time was six months, but VAS timing was not specified. Similar to the other study in which external neurolysis alone was performed, nine of the 11 patients (81.8%) in the Kibici et al. study experienced worsening of motor strength in their wrist and finger flexor and extensor muscles at their six-month follow up, whereas the remaining two patients maintained equivalent hand muscle strength pre- and post-operatively. Finally, in the third study of four patients who underwent external neurolysis alone, outcomes were mixed. Three patients (75%) experienced no change in motor deficits post-operatively, while one patient (25%) experienced diminished motor deficits. Two patients (50%) reported no change in sensory symptoms, while one patient (25%) experienced transient improvement and the fourth patient (25%) reported lasting improvement in sensory deficits. Finally, two patients (50%) did not experience any improvement in pain post-operatively, while one patient (25%) experienced transient reduction in pain and one patient (25%) experienced permanent reduction in pain. The patient with temporary improvement in sensory deficits and pain experienced subsequent deterioration over a two-year period requiring repeat surgical exploration.

In the case report detailing a patient who underwent brachial plexus exploration, neurolysis, and transfer of one ulnar nerve fascicle to the biceps brachii, the patient experienced a substantial improvement in neuropathic pain within one week of surgery. The patient’s elbow flexion improved from limited biceps brachii power preoperatively to biceps power M4 (against resistance) and a positive Tinel’s sign at the level of the biceps post-operatively. The patient also experienced an improvement in the ability to abduct her shoulder joint to 45° (supraspinatus and deltoid power M3), both of which were Medical Research Council (MRC) grade 0 preoperatively. The patient’s intrinsic hand muscles exhibited good motor function post-operatively and normal 2-point discrimination in the small finger (4 mm). Nerve conduction studies confirmed reinnervation of the affected biceps muscle. While sensory changes were not described in detail for this patient, the report notes that pre-operative C6–C7 dermatomal numbness persisted. Despite this, the patient experienced complete resolution of neuropathic pain with no ulnar nerve deficit.

In the study detailing a patient who underwent internal neurolysis and double fascicular transfer of median and ulnar nerves, the patient experienced reinnervation of her elbow flexors post-operatively, achieving MRC grade 4/5 elbow flexion strength after two years (improved from no active elbow flexion preoperatively). The patient reported good hand function, sensation and strength post-operatively. While sensory changes were not described in detail, the patient’s pain improved significantly, and the report states that the patient remained pleased with her outcome.

The patient who underwent gracilis-free muscle transfer experienced a mild improvement in symptoms [8]. 24 months post-operatively, the amplitude of motor unit potentials increased to 4 mV compared to prior denervation potentials, and the patient was able to flex the elbow 40–110 degrees, improved from grade 0 elbow flexion preoperatively. The patient also had full finger extension in 10-degree wrist flexion and was able to flex the fingers, improved from the patient’s preoperative state of weak finger extension (MRC grade 2). After surgery, the patient was able to voluntarily grasp and hold small objects and perform activities of daily living, such as holding objects down while cutting with a knife held in the contralateral hand. However, shoulder active range of motion remained unchanged post-surgery and there was no improvement in neurologic symptoms. Of note, the patient’s Disabilities of Arm, Shoulder, and Hand functional score improved from 56 to 21 [8]. Sensory and pain changes were not described.

One patient who underwent external neurolysis with subsequent omentum flap coverage did not experience significant improvement. Motor strength remained equivalent to preoperative strength in elbow flexion (grade 0) and arm adduction (grade 3). Arm abduction, elbow extension, and hand muscle strength worsened post-operatively [13]. The patient was LENT-SOMA grade 4 preoperatively and LENT-SOMA grade 2 post-operatively. At the 6-month post-operative visit, the patient was still experiencing paresthesias in the hand, including tingling in the lateral palmar region and dorsal region of the hand as well as the lateral areas of the upper arm and forearm. While motor function worsened and sensory function remained equivalent, the patient’s lymphedema improved and neuropathic pain achieved a VAS score of 0/10 (from 10/10 preoperatively) [13].

Segmental nerve resection and autografting led to improvements in antigravity elbow flexion that were assessed by improved biceps muscle strength in 6/6 patients (100%). Specifically, the mean muscle strength of biceps increased from 0.17 preoperatively to 3.5 post-operatively. Five patients (83.3%) experienced improvement in active range of motion with movement of elbow extension to flexion, and one patient (16.7%) experienced improvement in active range of motion with movement of elbow extension to flexion and shoulder adduction to abduction. Sensory changes were not described. Similar to motor changes, pain improved in three patients (3/6; 50%), and from the study sample of six patients, the mean VAS for pain improved from 2.7 preoperatively to 0.7 postoperatively over the mean follow-up period of 33 months. Two (2/6; 33.3%) of patients were not experiencing pain prior to surgery and thus experienced no change in VAS. One patient (1/6; 16.7%) did not experience improvement in pain. It is important to note that two of the patients in this study had previously undergone neurolysis for their symptoms, both of whom underwent prior neurolysis surgeries at a different hospital than the autografting surgery [12]. In both cases, neurolysis led to worsening symptoms. In one case, prior neurolysis surgery was followed by complete loss of function of shoulder abduction and elbow flexion [12]. In the second case, neurolysis had been performed twice prior to the autograft surgery, the first time with neurolysis of the supraclavicular brachial plexus four years prior and the second time with neurolysis of the brachial plexus and latissimus dorsi transposition three years prior [12]. There was no improvement after these surgeries and muscle strength deteriorated [12].

Finally, the five patients who underwent cervical thoracic laminectomy and microsurgical dissection of the dorsal root entry zone followed by dorsal root entry zone lesioning experienced strong pain outcomes [14]. Motor and sensory post-operative changes were not described. After the procedure, all patients (5/5; 100%) reported nociceptive pain at the cervical incision site, which was controlled with analgesics and resolved [14]. Thereafter, all five patients experienced an improvement in pain symptoms; three patients (3/5; 60%) experienced complete relief, one patient (1/5; 20%) experienced moderate relief of symptoms, and one patient (1/5; 20%) experienced complete relief after additional lesions [14].

### Surgical Complications

Explicit mention of complications was only seen in two studies, one of which stated that no surgical complications were noted [12]. In the study in which dorsal root entry zone lesions was used, adverse effects were seen in three out of five (3/5; 60%) patients [14]. One patient (1/5; 20%) experienced temporary lower limb ataxia lasting six weeks, while another patient (1/5; 20%) experienced transient urinary retention lasting three days [14]. A more serious complication was seen in one patient (1/5; 20%) who experienced worsening lower limb paresis over a follow-up period of 21 months [14]. Two other studies did not mention complications explicitly, however in the study by De Oliveira et al. in which neurolysis with omentoplasty was performed, the patient experienced worsening of motor strength—arm abduction, elbow extension, and intrinsic muscles of the hand, postoperatively. Similarly, in the Gosk et al. study in which external neurolysis alone was used, one of the four patients (1/4; 25%) had improvement in symptoms over a two-year period followed by deterioration requiring repeat exploration of the brachial plexus, which provided only temporary improvement. Remaining studies, while not mentioning explicit complications, listed the irreversible nature of surgery and the use of surgery only as a refractory treatment option.

Characteristics of studies as well as major study findings can be found in Tables 1 and 2.

## Discussion

Up to 50% of patients with breast cancer undergo radiation therapy, owing to radiotherapy’s crucial role in the comprehensive treatment of breast cancer, reducing local cancer recurrence and increasing overall survival [17–19]. Delivered intra- or post-operatively, radiotherapy typically targets the breast and lymph nodes. Radiation therapy carries several risks including secondary malignancies, heart failure, and lymphedema. Advances in radiation have lowered complication rates, yet brachial plexus neuropathy persists as a severe side effect causing functional limitations and chronic pain [3, 6].

Studies have found varied RIBPN incidence rates in radiated patients. The initial report documenting the phenomenon of RIBPN in breast cancer patients showed a 15% prevalence among individuals receiving a 51 Gy dose of therapy and 73% at 55 Gy dose [1, 20]. More recent findings suggest 1–9% prevalence in those treated with radiation to the supraclavicular and axillary regions [7, 8]. However, comprehensive data on RIBPN’s true incidence and prevalence are sparse. Many studies overlook RIBPN, focusing instead on radiation side effects of frozen shoulder and lymphedema. RIBPN’s underdiagnosis may stem from lack of awareness, symptom reporting delays, diagnostic challenges, or a lack of specialized care [9]. As survivorship continues to increase among breast cancer patients, the number of patients affected by RIBPN is expected to increase [21].

Brachial plexus neuropathy results from radiation-induced damage to the nerve network supplying the upper limbs, leading to symptoms like pain, weakness, and numbness [22]. This occurs when radiation causes localized ischemia and failure of cell proliferation, resulting in fibrosis of neural and perineural soft tissues [5]. More specifically, the pathophysiology of RIBPN involves a multifactorial process affecting both neural and vascular components of the brachial plexus. First, ionizing radiation directly damages DNA and cellular structures within neurons, Schwann cells, and surrounding tissues [5, 15]. This injury sparks a chronic inflammatory response with the activation of fibroblasts and the release of profibrotic cytokines such as transforming growth factor-beta (TGF-β) [5, 9, 15]. Over time, this process leads to extensive collagen deposition and fibrosis in the perineural and endoneural spaces, which results in mechanical compression of nerve fibers and impaired axonal transport [5, 9]. At the same time, radiation damages the microvasculature supplying the brachial plexus through endothelial injury, capillary rarefaction, and occlusion of small blood vessels [5, 9]. This may result in chronic ischemia, contributing to axonal degeneration and demyelination [9]. The combination of ischemic injury, chronic inflammation, and fibrosis creates a hostile environment for neural function and repair [5]. The most common post-radiation symptoms observed were neurogenic pain, hypoesthesia, and weakness in all studied patients, with 63.4% experiencing distal muscle weakness affecting the hand and 4.9% showing significant loss of grip strength, affecting daily activities. Muscle cramping was also noted as a less common symptom.

Satisfactory treatments for brachial plexus injury from radiation therapy are limited [23]. Medical treatments, while well-documented, often prove ineffective in advanced disease and lead to adverse drug effects. Therefore, for severe cases or when conservative treatment fails to alleviate symptoms, surgical interventions are considered. There have been a few studies that have discussed surgical management of RIBPN [3, 5, 11, 24]. Reviewed studies highlighted six surgical techniques for RIBPN, including various forms of neurolysis and nerve grafting, with outcomes categorized into motor recovery, sensory changes, and pain management (Table 3).

The most used surgical technique for RIBPN was found to be neurolysis alone or used with other surgical procedures. Neurolysis involves removing or releasing scar tissue or other obstructions compressing the nerve and can be performed internally by dissecting the nerve or externally by relieving compression of the nerve [25]. Both methods can be combined with other surgical techniques to alleviate tension on the nerve and restore nerve function [26, 27].

Despite the current belief that neurolysis provides the strongest surgical option for RIBPN, results from our review indicate that neurolysis may not be the most effective treatment for these patients. Only 3.8% of patients who underwent neurolysis alone saw significant motor improvements; 11.5% had mild motor improvements; and another 11.5% saw improvements at the shoulder. Sensory symptoms improved significantly in 38.5% of patients and an additional 11.5% of patients experienced a mild improvement in sensory deficits (from S0 preoperatively to S1 post-operatively), while 50% of patients had no sensory Changes post-operatively. Perhaps the most promising effect of neurolysis surgery was on pain reduction, with 80.8% of patients experiencing a significant improvement pain, while 19.2% of patients experienced persistence of pain symptoms.

These results highlight important limitations surrounding the efficacy of neurolysis outside of pain relief, which may be attributable to the nature of the intrinsic nerve damage caused by radiation [9]. Additionally, neurolysis carries the risk of exacerbating nerve ischemia, potentially leading to intractable pain or permanent sensory and motor deficits. While these outcomes were not seen in this review, they have been described in prior studies investigating neurolysis and should be considered given the devastating consequences for patients [28].

Neurolysis with nerve transfer was described in two case reports. Nerve transfer is a technique used to restore function to muscles that have lost nerve supply [29–31]. Nerve transfer involves connecting a donor nerve to a recipient close to the paralyzed muscle, providing a nearly immediate source of innervation. This technique has revolutionized the treatment of severe nerve injuries, especially in cases of brachial plexus damage [29, 31].

This systematic review includes two case reports on neurolysis and nerve transfer for RIBPN, highlighting its potential for improving motor function. In one report, a patient underwent neurolysis, scar tissue release, and double fascicular transfer using median and ulnar nerves. Another patient experienced full brachial plexus exploration and single ulnar nerve fascicle transfer to the biceps brachii nerve. Despite varying techniques, both patients showed significant post-operative motor improvements, especially in elbow flexion and biceps strength, and reported significant pain relief, with one achieving complete pain resolution. These results suggest neurolysis followed by nerve transfer could be effective for motor restoration and pain relief in RIBPN, meriting further investigation despite limited details on sensory outcomes.

Gracilis-free functional muscle transfer (FFMT) was used in one case report to restore muscle function including elbow flexion and to optimize hand function [32]. In this case, neurolysis was performed prior to muscle transfer. The gracilis muscle was selected due to its long tendinous portion that can be sutured to the finger extensors [8]. By two years post-surgery, the patient regained elbow range of motion from 40 to 110 degrees and had strengthened hand function; She was able to voluntarily grasp, hold small objects and perform activities of daily living. The improvement in the Disabilities of Arm, Shoulder, and Hand score from 56 to 21 post-operatively highlighted the treatment’s effectiveness in motor recovery. FFMT is thus considered a valuable adjunct in treating RIBPN.

Omentoplasty, a procedure in which the omentum is used to repair or reinforce damaged tissues, has also been evaluated for treating RIBPN. The omentum, rich in blood supply from fatty abdominal tissue, is favored for reducing edema and promoting neovascularization [33]. In one case study, neurolysis with omentoplasty showed mixed results: motor strength was largely unchanged or worsened in some movements; while the patient’s motor strength was equivalent to preoperative strength in arm adduction (grade 3) and elbow flexion (grade 0), it worsened (grade 2) in arm abduction, elbow extension, and the intrinsic muscles of the hand, decreasing from LENT-SOMA grade 4 to grade 2 post-operatively. Pain significantly decreased post-surgery, achieving VAS 0/10 (from 10/10 preoperatively) and driving a progressive opioid withdrawal. Sensory symptoms remained stable, but the patient’s lymphedema improved, suggesting benefits for patients with severe pain and lymphedema despite limited impact on motor and sensory functions (Table 4).

**Table 4:** Complications of surgical treatments

Intervention	Total number of patients	Complications
Neurolysis alone	26	1 patient had improvement in symptoms over a two-year period followed by deterioration requiring repeat exploration of the brachial plexus, which provided only temporary improvement. Otherwise, no complications mentioned
Neurolysis and nerve transfer	2	Not mentioned
Neurolysis and free functional muscle transfer	1	Not mentioned
Neurolysis with omentoplasty	1	1 patient experienced worsening of motor strength – arm abduction, elbow extension, and intrinsic muscles of the hand, postoperatively
Nerve grafting	6	Not mentioned
Dorsal root entry zone lesion (DREZ)	5	Adverse outcomes in 3/5 patients: 1 patient experienced temporary lower limb ataxia lasting six weeks, 1 patient experienced transient urinary retention lasting three days.1 patient experienced severe complication of worsening lower limb paresis over a follow-up period of 21 months

Six patients underwent nerve autografting in which healthy autologous tissues, including small nerves, muscle and vessels, are harvested and transplanted to a recipient site where damaged nerves are located [34, 35]. There, the fibers regenerate [35]. The sural nerve is often chosen because of its length, accessibility, reliable anatomic location and minimal donor site morbidity [36–38].

The study on nerve grafting demonstrated the effectiveness of this technique in treating motor and pain symptoms of RIBPN. All patients improved in bicep muscle strength, going from a mean of M = 0.17 preoperatively to M = 3.5 post-operatively. Additionally, 83.3% improved in active range of motion from elbow extension to flexion, and 16.7% saw improvements in active range of motion at the elbow and shoulder. Pain improved post-surgery for 50% of patients, with VAS dropping from 2.7 to 0.7. No sensory details were provided, and 33.3% had no pre-surgery pain, so their VAS remained unchanged.

Five patients underwent dorsal root entry zone (DREZ) lesions. The DREZ procedure involves a cervical thoracic laminectomy and dissection of the dorsal root entry zone, followed by creating radiofrequency lesions, thereby inactivating neurons in the dorsal horn [14]. Teixeira et al. reported 60% of RIBPN patients had complete pain relief and 20% experienced moderate relief with DREZ, showing its effectiveness against neuropathic pain.

As with any surgery, RIBPN surgeries have complications as well. Few of the reviewed studies address these complications comprehensively. In one study, omentoplasty led to worsened motor function including arm abduction, elbow extension, and hand muscle strength post-surgery [39, 40]. Despite this, neurolysis with omentoplasty is still preferred over neurolysis alone. In one study in which neurolysis alone was performed, 25% of patients initially improved, only to worsen over two years, necessitating additional surgeries with temporary relief. More severe complications were observed with DREZ surgery, including temporary lower limb ataxia, transient urinary retention, and exacerbated lower limb paresis. With these risks, surgical treatment is generally advised only when medical treatments fail [5]. It is important to note that, given the small number and heterogeneity of studies identified, formal subgroup and sensitivity analyses were not feasible.

## Conclusion

RIBPN is a debilitating complication of postoperative radiation therapy in breast cancer patients. Our literature review revealed that surgical treatment with neurolysis alone, neurolysis with nerve transfer, free flap muscle transfer, neurolysis with omental flap, nerve grafting or dorsal root entry zone lesions provide options for these patients, however, these modalities are not well studied. Further investigation into the efficacy of these surgical options, as well as alternative surgical treatments for RIBPN, may reduce morbidity and improve outcomes among breast cancer patients.

## Supplementary Information

Below is the link to the electronic supplementary material.

Supplementary file1 (DOCX 14 KB)

Supplementary file2 (DOCX 16 KB)

## Data Availability

This study is a systematic review of published literature. All data analyzed during this study were obtained from publicly accessible databases, including PubMed, SCOPUS, and Embase. The datasets supporting the findings of this review are available from the corresponding author upon request. No new or proprietary data were generated in the course of this research.
