# Supplementary material for: Surgical Treatment of Radiation-Induced Brachial Plexus Neuropathy in Breast Cancer Patients after Adjuvant Radiotherapy: A Systematic Review
Source: Indian J Orthop. 2025 Sep 8;60(3):741–70. doi: 10.1007/s43465-025-01540-0 (PMC13031617; doi:10.1007/s43465-025-01540-0)
Supplement: Supplementary file 1 — Supplementary file1 (DOCX 14 KB) [file 43465_2025_1540_MOESM1_ESM.docx]

**SUPPLEMENTAL FIGURE 1.** Search terms and databases searched

| Database | Search terms |
| --- | --- |
| PubMed  Embase  SCOPUS | “radiation neuritis breast”  “radiation-induced brachial plexopathy”  “surgical treatment of radiation neuritis breast” and “surgical treatment of radiation-induced brachial plexopathy”  Restricted to 1/2005 – 4/2023 |
