# Supplementary material for: Surgical Treatment of Radiation-Induced Brachial Plexus Neuropathy in Breast Cancer Patients after Adjuvant Radiotherapy: A Systematic Review
Source: Indian J Orthop. 2025 Sep 8;60(3):741–70. doi: 10.1007/s43465-025-01540-0 (PMC13031617; doi:10.1007/s43465-025-01540-0)
Supplement: Supplementary file 2 — Supplementary file2 (DOCX 16 KB) [file 43465_2025_1540_MOESM2_ESM.docx]

**Supplementary Material: ROBINS-I Risk of Bias Assessment**

| **Study** | **Confounding** | **Selection** | **Intervention Classification** | **Deviations from Intended Interventions** | **Missing Data** | **Outcome Measurement** | **Selective Reporting** | **Overall Risk of Bias** |
| --- | --- | --- | --- | --- | --- | --- | --- | --- |
| Yin et al. (2023) | Serious | Moderate | Low | Low | Low | Serious | Moderate | Serious |
| Warade et al. (2019) | Serious | Moderate | Low | Low | Low | Serious | Moderate | Serious |
| de Oliveira et al. (2020) | Serious | Serious | Low | Low | Low | Serious | Moderate | Serious |
| Tung et al. (2009) | Serious | Serious | Low | Low | Low | Serious | Moderate | Serious |
| Gangurde et al. (2014) | Serious | Serious | Low | Low | Low | Moderate | Moderate | Serious |
| Teixeira et al. (2007) | Serious | Low | Low | Low | Low | Serious | Moderate | Serious |
| Kibici et al. (2020) | Serious | Low | Low | Low | Low | Serious | Moderate | Serious |
| Wong et al. (2009) | Serious | Serious | Low | Low | Low | Moderate | Moderate | Serious |
| Gosk et al. (2007) | Serious | Low | Low | Low | Low | Serious | Moderate | Serious |

All included studies were assessed as having a serious overall risk of bias, mainly due to serious concerns in confounding and outcome measurement domains. The limitations arise primarily from the observational designs, absence of control groups, and lack of blinding in outcome assessment. These findings indicate caution in interpreting efficacy outcomes from these studies.
